# Designing for Impact and Institutionalization: Applying Systems Thinking to Sustainable Postpartum Family Planning Approaches for First-Time Mothers in Bangladesh

**DOI:** 10.9745/GHSP-D-22-00023

**Published:** 2022-10-31

**Authors:** Melanie Yahner, Angela Muriuki, Amy Mangieri, Syeda Nabin Ara Nitu, Shumona Shafinaz, Eric Sarriot

**Affiliations:** a Save the Children US, Fairfield, CT, USA.; b Independent consultant; formerly of Save the Children Kenya, Nairobi, Kenya.; c Save the Children US, Washington, DC, USA.; d Save the Children Bangladesh, Dhaka, Bangladesh.; e Independent consultant; formerly of Save the Children Bangladesh, Dhaka, Bangladesh.; f Gavi, the Vaccine Alliance, Geneva, Switzerland; formerly of Save the Children US, Washington, DC, USA.

## Abstract

Integrated service delivery approaches have shown promise to increase use of services including postpartum family planning (PPFP) by young, first-time mothers (FTMs) but have proven challenging to scale and institutionalize. Integration adds complexity, requiring careful assessment of effects on a range of key system functions from demand creation and service delivery to oversight and governance. Through an innovative design process, we selected approaches to increase FTMs’ PPFP use through existing health systems. We generated programmatic options and then sought to select approaches based on (1) potential impact on FTMs’ PPFP uptake and (2) potential to institutionalize in the health system. The latter represented an innovation in addressing management systems’ drivers of scalability and sustainability; to accomplish it, we developed a participatory design process to assess the potential of an approach to be institutionalized in a specific context.

We adapted a management systems theory, the Viable System Model (VSM), which presents 5 essential organizational functions and the relations required between them to improve the viability (performance and institutionalization) of organizational systems. Drawing from the VSM, we developed a process for reviewing the effects of proposed approaches on provider workload, client flow, infrastructure, revisions to guidelines and job descriptions, coordination and management, and information systems. The VSM provided a structure to identify potential displacement of capacity in the health system and mitigate often neglected organizational challenges that compromise institutionalization. The process informed the elimination of approaches with potential for impact but that had deal-breakers to institutionalization, such as increased workload or shifted job descriptions, in the Bangladeshi context. For the selected approaches, consideration of systems elements fostered discussion of expected risks to institutionalization, highlighting needed mitigation efforts and monitoring during implementation.

## Abstract (precis)

Public health practitioners often design interventions prioritizing potential impact over sustainability. To assess the potential for impact and institutionalization, we applied systems thinking to postpartum family planning approaches for first-time mothers in Bangladesh.

## Key Messages

- Donor-funded multilevel approaches to address barriers for young first-time mothers in using modern postpartum family planning methods have largely focused on integrated service delivery—an approach that has proven challenging to institu-tionalize within government health systems.
- To achieve lasting impact, an approach must be both effective (able to produce the desired impact) and able to be institutionalized. Yet, considerations for whether and how approaches can be institutionalized within existing health systems are often underrepresented in program design.
- To increase the use of postpartum family planning by first-time mothers in Bangladesh, we adapted the Viable System Model to the context of the Bangladeshi health system and developed a tool and process for assessing the potential for institutionalization alongside potential for impact during a program design process.

## INTRODUCTION

The youngest mothers and their babies have increased risk of poor maternal and infant outcomes.^1^^,^^2^ In many settings, adolescent mothers are most likely to have closely spaced subsequent pregnancies,^3^ which further increases risk.^4^ Increasingly, the transition to becoming a parent for the first time is seen as a window of opportunity to shape lifelong practices. These include modern postpartum family planning (PPFP) use during the 12 months after childbirth, an important contribution to healthy timing and spacing of subsequent pregnancies.^5^

A growing body of program efforts have sought to address the supply- and demand-side barriers to PPFP faced by young, first-time mothers (FTMs; aged 15–24 years). In many low- and middle-income countries (LMICs), family-, community-, and health system–level factors shape FTMs’ PPFP use.^6^^,^^7^ To date, most efforts targeting FTMs have entailed multilevel approaches coupling integrated service delivery, including fostering respectful care, with community-level interpersonal communication efforts to address normative and social factors that limit service use.^8^^,^^9^

Despite the promise of effectiveness, complex, multilevel FTM initiatives have proven challenging to fully scale and institutionalize.^10^ This challenge is hardly unique; other interventions with demonstrated impact at small scale have failed to produce the same results when scaled beyond small geographic areas^11^ or have proven infeasible to institutionalize.^12^^,^^13^ For sustainability beyond donor-funded projects, practitioners must “begin with the end in mind”^14^ by considering and accounting for how health systems’ characteristics may facilitate or impede institutionalization.

Most efforts targeting FTMs’ PPFP use have entailed complex, multilevel approaches coupling integrated service delivery with community-level interpersonal communication efforts.

The field of systems thinking has increasingly gained recognition among researchers and practitioners as offering frameworks through which to understand and manage organizational complexity, such as that of a health system.^15^ One systems thinking approach, the Viable System Model (VSM), provides a general theory to consider organizational functions and structures required to deliver services and remain viable. Viable organizations are “capable of adapting appropriately to their chosen environment, or adapting their environment to suit themselves.”^16^^–^^18^ With principles applicable to any organization, the VSM is sector agnostic and posits that a set of 5 recursive systems are critical to performance. From individual units to the highest organizational levels, these systems encompass operations, coordination, and 3 different levels of management. Health information systems support operations management. We refer to the VSM systems as functions, which better fits our global health culture. The VSM provides rules and principles for managing complexity, which increases as organizations expand in mission, resources, and size, and can create confusion, gaps in performance, loss of efficiency, and failure to sustain change.

Despite growing recognition of the need to consider the complexity of health systems beyond static elements, or building blocks,^19^^,^^20^ few practical approaches are available to guide practitioners to explore the potential of an approach to be institutionalized—embedded within budgets, plans, frameworks, and policies, and routinized in services and processes—in dynamic, complex health systems.^15^

### The Connect Project

Led by Save the Children with funding from the Bill & Melinda Gates Foundation, the Connect project (2019–2024) aims to develop sustainable PPFP approaches for FTMs in Bangladesh and Tanzania through existing large-scale, donor-funded projects already supporting primary health care platforms. Having worked on the project, we chose to focus on Bangladesh to provide an in-depth exploration of the experience there. In 2020, the project design team, comprising project technical and management colleagues, national health system stakeholders, and researchers, and led by global and country experts, formulated effective, scalable PPFP integration approaches. Approaches were required to show (1) potential for impact with the target population, based on global evidence and formative research; and (2) potential for institutionalization, based on organizational management conditions of the health system. In this article, we detail a practical method for designing integration approaches with potential for both impact and institutionalization, applied to the design of approaches to increase PPFP use among FTMs in Bangladesh.

We detail a practical method for designing integration approaches with potential for both impact and institutionalization.

### Family Planning in the Bangladesh Context

In Bangladesh, while median birth intervals are relatively long at 55.7 months, mothers aged 15–19 years have significantly shorter intervals compared to mothers aged 20–29 years (25.3 months vs. 49.4 months).^21^ Modern family planning (FP) use among all married women is relatively high at 51.9%, but those aged 15–19 years have lower FP use (43.7%). Girls and younger women are more likely to use short-acting FP methods, with 95.7% of 15- to 19-year-old and 93.5% of 20- to 25-year-old modern FP users using pills, condoms, or injectables. Importantly, 37.1% of all married women discontinue FP use within 12 months of adoption.

Within the Ministry of Health and Family Welfare, the Directorate General of Health Services and the Directorate General of Family Planning oversee separate facility- and community-level health facilities and provider cadres. The Government of Bangladesh had made strong commitments to PPFP, as demonstrated in its FP2020 commitments.^22^

## METHODS

**FIGURE 1:**
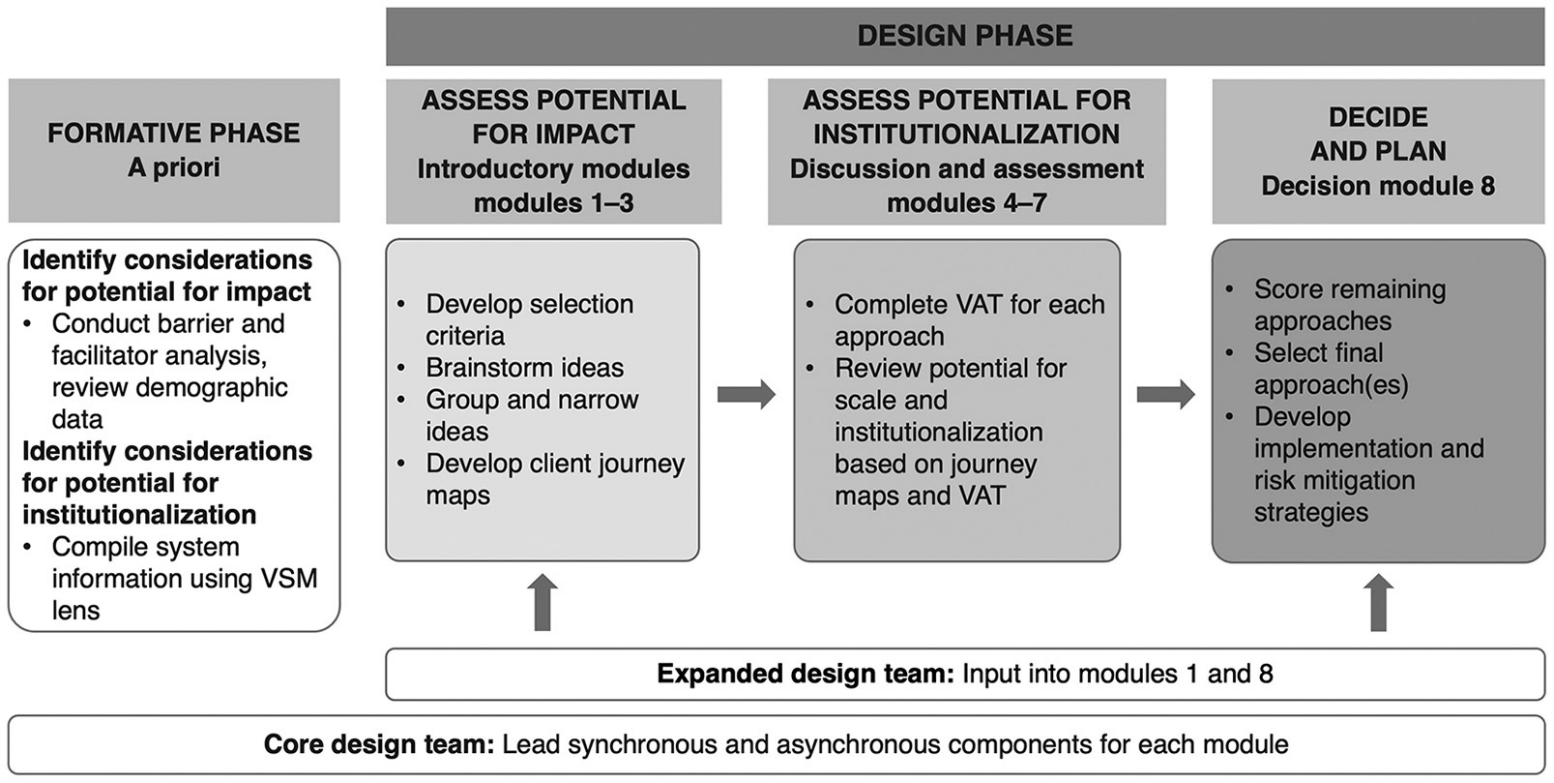
Sequence of Formative and Design Phases for Designing Sustainable PPFP Approaches for FTMs Abbreviations: FTM, first-time mother; PPFP, postpartum family planning; VAT, Viability Assessment Tool; VSM, Viable System Model.

Our process for designing sustainable FTM approaches entailed conducting formative research, ideating approaches for integrated PPFP service provision targeting FTMs, and assessing potential for institutionalization. We outline each step, detailing the assessment of potential for institutionalization as an innovation, which is underrepresented in many design processes. With the start of the coronavirus disease (COVID-19) pandemic coinciding with the design onset, our process had to avoid travel and large gatherings. Figure 1 outlines the formative and design phases, which are detailed in the following sections.

### Formative Phase

#### Identify Considerations for Potential for Impact

We conducted formative research and reviewed demographic evidence to identify key barriers to FTMs’ PPFP use.

We led a formative barrier and facilitator analysis^23^ in 2 upazilas of Noakhali district in Chattogram division, using participatory qualitative methods to identify factors influencing FTMs’ use and nonuse, and delayed initiation and discontinuation, of reproductive, maternal, newborn, and child health (RMNCH) services including PPFP, as well as to identify factors influencing health system responses to FTMs’ needs. The analysis used a socioecological approach,^24^ sampling 20 FTMs, 10 male partners, 4 facility-based providers, and 14 community-based providers. The core design team synthesized findings, pinpointing the key barriers to PPFP use that the approach would need to address. We also reviewed secondary data analyses of the 2016 Bangladesh Demographic and Health Surveys and 2019 Multiple Indicator Cluster Survey to identify RMNCH services through which Bangladeshi mothers aged 15–24 years interacted with the health system during pregnancy and the postpartum period.^25^^,^^26^

#### Identify Considerations for Potential for Institutionalization

Using context experience and expertise, Bangladeshi Connect project staff compiled basic information about the health system organization and delivery of RMNCH services. Using a simple checklist probing each of the VSM functions, they documented the number and type of health facilities, facility- and community-based providers by cadre, client flows for each RMNCH service, supervisory roles and mechanisms, community health worker tasks and client loads, and community groups involved in health promotion.

### Design Phase

The design phase encompassed 8 sequential modules (Figure 1), with 1 module completed per week. For each module, a core design team (Box 1) engaged stakeholders through synchronous video meeting components interspersed with asynchronous work (e.g., background reading, follow-up emails, and smaller group discussions).

BOX 1 **Design Team Composition**

The core design team included 6 Connect project staff:

Bangladesh (3)

- Project lead with expertise in adolescent health (1)
- Technical experts:
  - Maternal and newborn health (1)
  - Monitoring and evaluation (1)

Global (3)

- Project director with expertise in adolescent health (1)
- Technical experts:
  - Health systems strengthening and systems thinking (1)
  - Maternal and newborn health (1)

At key decision points, the expanded design team, which included 20 national stakeholders outside of the core team and 3 U.S.-based program evaluators, provided input into the conceptualization and selection of the approaches.

- Ministry of Health and Family Welfare (8)
  - Directorate General of Family Planning (7)
  - Directorate General of Health Services (1)
- Nongovernmental organizations (7)
- Private sector service delivery organizations (4)
- Professional association (1)
- Global evaluation and research experts (3)

#### Assess Potential for Impact: Modules 1–3

**Develop Selection Criteria A Priori.** In the opening modules, a member of the core design team with expertise in systems thinking provided an orientation to systems thinking and to the VSM for the expanded design team.

Drawing from the VSM and considering ExpandNet^27^ scalability questions, the core design team developed 12 a priori criteria for selecting the final approach later in the process (Module 8). The criteria encompassed both potential for impact with FTMs and potential to be institutionalized into the Bangladesh health system (Supplement 1).

**Brainstorm Ideas With Potential for Impact.** The core and expanded design teams reviewed barrier and facilitator analysis findings, focusing on key barriers to FTMs’ PPFP use. Drawing from collective expertise, we brainstormed 54 approaches that had the potential to increase PPFP uptake among FTMs in Bangladesh by addressing barriers identified in the formative stage.

**Group and Narrow Ideas.** The core design team subsequently narrowed the list significantly by grouping duplicative ideas (18 ideas) and removing those that may have contributed to improving PPFP uptake as part of a comprehensive approach but did not address barriers on their own (e.g., quality improvement efforts, strengthening referral systems), or did not address a clear barrier to FTMs’ service use (e.g., a mobile hotline) and thus presented limited impact potential (16 ideas removed). We clustered the remaining 20 ideas by the entry point at which FTMs would be reached for the first time (i.e., during antenatal care [ANC] [6 ideas], during intrapartum or predischarge care for those delivering in facilities [4 ideas], through immunization services [4 ideas], through special care for small and sick newborns [2 ideas], or at the community level [4 ideas]). The core design team then prioritized 1 idea from each entry point for further discussion. The team based these decisions on consideration of potential for impact and feasibility of implementation, as informed by existing evidence, and technical and context experience and expertise. By the conclusion of this short-listing exercise, 5 approaches remained for further examination and consideration.

**FIGURE 2:**
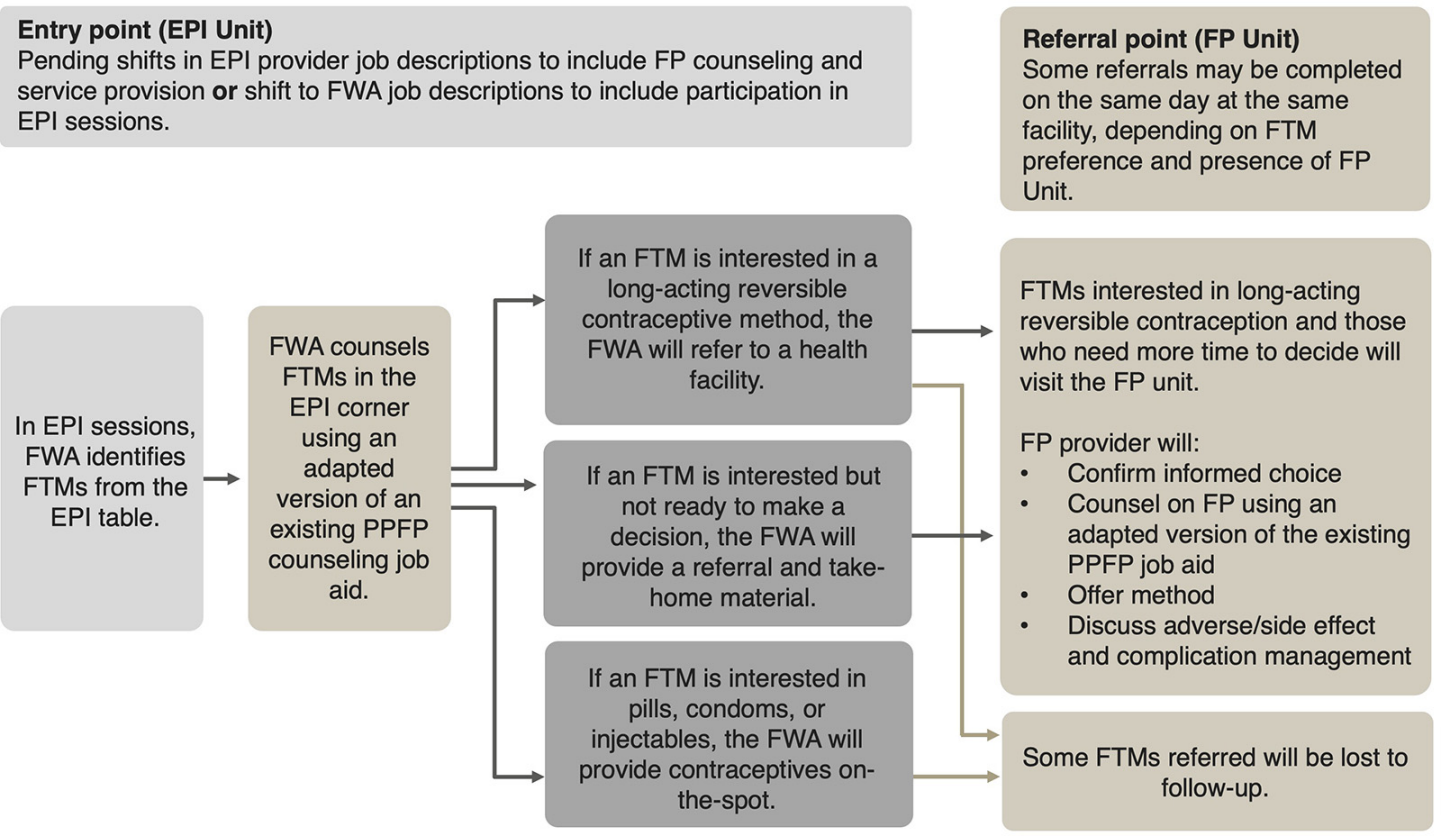
Illustrative Client Journey Map for Integrated PPFP/Immunization Approach That Was Excluded Abbreviations: EPI, Expanded Programme on Immunization; FP, family planning; FTM, first-time mother; FWA, family welfare assistant; PPFP, postpartum family planning.

**Develop Client Journey Maps.** For each of the 5 remaining ideas, through a collaborative asynchronous approach, the core design team developed a visual client journey map depicting the entry point where facility- or community-based providers would interact with FTMs to provide information and/or services, referral point(s) where providers may send FTMs for information, services, and/or follow-up, and needed coordination between service delivery points. These journey maps depicted a future state in which all needed functions would be established and operational (e.g., adequate staffing and established referral mechanisms). Figure 2 depicts an illustrative client journey map for 1 approach assessed.

#### Assess Potential for Institutionalization: Modules 4–7

We adapted the VSM to the health system context to assess the potential for institutionalization of approaches (Table 1). This informed our understanding of how the potential approaches’ viability could be affected by the health system characteristics and potential displacement within the health system (i.e., saturating the capacity of existing human resources, such as increased workload or shifted client flow, or displacing priorities to the detriment of other essential services). We referred to insurmountable displacement as a deal-breaker throughout the design process. Supplement 2 details the VSM and its application to health systems.

**TABLE 1.** Health System Functions Drawn From the VSM, With Illustrative Assessment Areas^a^

**Functions and Corresponding VSM Systems**	**Illustrative Assessment Areas**	**Number of Questions in VAT **
Service delivery (System 1)	Staffing levels Motivation and skills of needed staff Inclusion of approach in existing staff job descriptions	3
Coordination (System 2)	Coordination of services between SDPs to ensure a full package of care How clients and information move between SDPs, within and outside the facility, and to community providers	7
Supervision, management, oversight, control (System 3)	Supervision tools for SDP and system managers Clarity of supervision approaches and processes Applicability of existing supervision procedures to the proposed approach	5
Higher-level management/planning; policy and governance (Systems 4 and 5)	Management structures in place (within SDPs, at the facility, and within the subnational unit) to ensure effective service provision Operationalization of national-level policies and standards at subnational, facility, and SDP units	2
Support systems: Supply chain	Systems supporting commodity and supply management	3
Support systems: Information systems	Availability and accessibility of information on service delivery, support systems, and operations to SDP (and system) managers How information is used in day-to-day and higher-level decisions	3

Abbreviations: SDP, service delivery point; VAT, Viability Assessment Tool; VSM, Viable Systems Model.

^a^ See Supplement 2 for further detail about the adaptation of the VSM to the health system context.

We adapted the VSM to the health system context to assess the potential for institutionalization of approaches.

**Complete Viability Assessment Tool.** To operationalize the VSM to the questions faced by the primary health care systems in Bangladesh in integrating PPFP into existing services, the core global project design team developed the Viability Assessment Tool (VAT) through an iterative process over Modules 4–7. The VAT probed organizational functions including service delivery interactions; coordination; supervision, management, oversight, and control; and systems for higher-level planning, policy and governance, support, and information. We considered information systems as a support system, alongside the supply chain support system; the VAT explored these as support systems to the delivery of client services. Under VSM principles, the performance and sustainability of these support systems must also meet conditions of viability.

The VAT used a client-centered approach following an FTM’s journey through the Bangladesh primary health care system, beginning from a facility or community entry point and encompassing needed referrals within and between service delivery points. Open-ended and closed questions guided Bangladesh-based members of the core design team to define intervention packages (e.g., tools, trainings) and consider the implications of each of the 5 short-listed approaches, guided by the VSM functions (Table 1). The VAT is provided as Supplement 3.

**Review Potential for Institutionalization.** Based on information recorded in the VAT, the core design team discussed the potential of each approach to be institutionalized. We chose not to use the VAT as a scoring or ranking tool but rather as a thinking tool to systematically identify strengths and weaknesses of the 5 final approaches (Table 2). We also reflected on the extent to which the functions depicted in the journey map for each potential approach were established and operational and what intervention would be needed to create the future state depicted in the journey map. We critically discussed to distinguish deal-breakers from risks amenable to mitigation. During the initial viability discussion, the core design team eliminated approaches found to have deal-breakers from further consideration, further narrowing the short list.

**TABLE 2.** Key Findings From Assessment of Potential for Institutionalization

**Approach**	**Immediate PPFP**	**Predischarge**	**Targeted Home Visits**	**KMC/SCANU**	**PPFP/EPI Integration**
Service delivery (System 1)	Most providers trained in PPFP and provide integrated services; few upskilling needs^a^	Most providers trained in PPFP and provide integrated services; few upskilling needs^a^ Limited observation and discussion during busy discharge times^b^ Increased workload^b^	FWA JDs included HVs^a^ High FWA vacancies, infeasible HV targets^b^	Nurses in KMC/SCANU not trained in PPFP^b^	No integration by EPI or FP providers^b^ Negative impact on provider workload^b^ Facility infrastructure unconducive to private FP counseling^b^
Coordination (System 2)	Existing monthly coordination meetings^a^ Needed coordination of counseling beginning from ANC, documenting and following up on clients’ PPFP intentions^b^	Existing coordination meetings^a^ Poor referral coordination^b^ Needed internal coordination during busy discharge times, support for additional tasks^b^	Existing coordination meeting of supervisors, service providers, and FWAs^a^ Needed coordination of facility referrals^b^	No existing coordination or referrals between KMC/SCANU and FP units^b^	Needed coordination to ensure FP providers present in EPI sessions^b^ Needed coordination of intra- and inter-facility referrals^b^
Supervision, management, oversight, control (System 3)	Existing supervision mechanisms^a^	Monthly supervision visits planned^a^ Little follow-up after supervision^b^	Monthly supervision visits planned^a^ Little supportive supervision, HVs rarely observed^b^	Monthly supervision visits planned^a^ PPFP not included in provider supervision^b^	Integrated services not included in supervision^b^
Higher-level systems (System 4)	National stakeholder commitment to PPIUD^a^ Existing (underutilized) fund to offset PPFP expenses^a^^,b^			Different managers in KMC/SCANU and FP units, increasing coordination needs^b^	Stakeholder commitment to FP/EPI integration^a^
Support systems/supply chain	FP commodity supply generally sufficient^a^			FP commodities would need to be supplied in units^b^	
Support systems/information systems	Existing processes to counsel and document PPFP preferences in ANC card^a^ Inconsistent documentation of PPFP preferences in ANC^b^	FTMs identifiable in delivery, MNC registers^a^ No system of documenting status at discharge^b^	FTMs identifiable in community pregnancy registration systems^a^ No mechanism prompted FWA HV after delivery^b^	FTMs not identifiable in KMC/SCANU registers^b^	FTMs identifiable in EPI registers or screening^a^ EPI and FP registers not linked or harmonized^b^
Decision	Combined as final selected approaches, with risk mitigation efforts			Determined to have deal-breakers in the context	

Abbreviations: ANC, antenatal care; EPI, Expanded Programme on Immunization; FP, family planning; FTM, first-time mother; FWA, family welfare assistant; HV, home visit; JD, job description; KMC, kangaroo mother care; MNC, maternal and newborn care; PPFP, postpartum family planning; PPIUD, postpartum intrauterine device; SCANU, Special Care Newborn Unit.

^a^Denotes opportunities.

^b^Denotes potential risks to viability based on current system characteristics.

#### Decide and Plan: Module 8

Lastly, the core design team selected the final approach in consultation with the expanded design team.

**Score and Select Approaches Against Selection Criteria.** Through a Google Forms survey with a Likert scale, the expanded design team scored each of the remaining approaches based on selection criteria determined a priori (Supplement 1) and discussed collaboratively. While we calculated mean scores for each approach, we did not simply select the highest-scoring approach. Rather, scores informed further discussion about whether low-scoring items constituted deal-breakers compromising viability, and how low-scoring items could be mitigated in implementation.

**Develop Risk Mitigation Strategies.** After selecting the final approaches, the core design team identified implementation strategies to proactively mitigate identified risks.

## RESULTS

### Formative Assessment Findings

Our formative assessment identified service use patterns, key barriers to FTMs’ PPFP use, and opportunities to reach FTMs with PPFP.

Our formative work identified service use patterns, key barriers to FTMs’ PPFP use, and opportunities to reach FTMs with PPFP.

#### RMNCH Service Use Patterns Among FTMs

Secondary analyses of Demographic and Health Surveys identified opportunities to reach FTMs by integrating PPFP into highly used services. Among mothers aged 15–24 years at the national level, used as a proxy for FTMs, ANC and immunization were the most utilized services; 86% made at least 1 ANC visit, often late in pregnancy, and 84% of babies born to mothers aged 15–24 years received 8 basic immunizations by age 2 years.^25^ Over half (56%) of mothers aged 15–24 years delivered at home,^26^ with many deliveries in private facilities. While 85% of all mothers delivering in a facility received postnatal care (PNC), just 42% of those delivering at home received PNC.

#### Factors Influencing FTMs’ RMNCH Service Use

The barrier and facilitator analysis pinpointed the following key factors influencing FTMs’ use and nonuse of RMNCH services. Mothers-in-law and male partners determined whether and where FTMs accessed RMNCH services; FTMs had limited negotiation power within family structures. Many PPFP adopters discontinued method use within 12 months due to side effects and pressure to become pregnant again. Mothers of all ages had increased responsibilities and restrictions on movement outside the home for 40 days postpartum due to religious and social prohibitions, which limited facility visits.

### Recommendations for Approach to Improve PPFP Use Among FTMs

The core design team determined that the selected approach(es) must address the above barriers by: reaching FTMs regardless of place of delivery; accounting for postpartum restrictions on movement; engaging family; addressing factors encouraging discontinuation; and integrating PPFP into other RMNCH touchpoints.

As described above, the expanded design team brainstormed 54 initial ideas with potential for impact (improved PPFP use among FTMs). The core design team subsequently narrowed a short list of 5 approaches for deeper discussion, developing 1 journey map for each to guide discussions. Approaches identified for deeper assessment, and the rationale from the perspective of impact, included:

- Strengthening immediate PPFP uptake through FTM-targeted FP counseling in ANC, early stages of labor, and immediately following delivery. Reaching FTMs with an integrated care package, leveraging higher ANC coverage, could provide multiple touchpoints for family involvement and offers the possibility of immediate PPFP.
- Strengthening predischarge counseling for FTMs in public and/or private facilities. This counseling, inclusive of PPFP, would reach FTMs delivering in health facilities, supporting early PPFP initiation and encouraging continuity of care.
- Supporting targeted home visits (HVs) to FTMs. HVs to pregnant and postnatal FTMs could reach FTMs regardless of place of delivery, engage family, and refer FTMs for facility-based PPFP.
- Strengthening PPFP counseling for FTMs with babies in kangaroo mother care units and special care newborn units. Adolescent FTMs are more likely to have preterm or low-birthweight babies.^29^ Mothers spend long periods of time in these units supported by families, providing opportunities for family engagement.
- Strengthening PPFP/immunization integration targeting FTMs. Integrating PPFP into the Expanded Programme on Immunization could leverage the service with the highest reach, engage FTMs regardless of place of delivery, and engage family. The potential of FP/immunization integration is well documented as a promising practice.^28^

### Viability Assessment Findings

We completed 1 VAT for each of these 5 approaches and discussed them among the core design team. The key findings from the assessment against the organizational functions that informed final decisions are presented in detail here and in Table 2.

We completed 1 VAT for each of the 5 approaches on the short list and discussed them among the core design team.

The core design team excluded 2 approaches from further consideration following the initial VAT completion, which identified the following deal-breakers (Box 2). After removing the first 2 approaches, the design team discussed the 3 remaining approaches in detail.

BOX 2 **Approaches Excluded That Had Deal-Breakers for Institutionalization**

**Strengthening PPFP Counseling for FTMs Through Kangaroo Mother Care and Special Care Newborn Units**

Facilities with kangaroo mother care units and special care newborn units did not include family planning (FP) services. The management and coordination burden of shifting provider job descriptions to provide FP counseling and methods in these units would be beyond the project’s scope. Further, some lower-level facilities served less than 10 mothers of all ages annually. We determined that the low potential to reach significant numbers of FTMs would not justify the needed management and coordination adjustments and risk of displacement.

**Strengthening PPFP/Immunization Integration Targeting FTMs**

In the Bangladeshi health system, 2 distinct directorates of the Ministry of Health and Family Welfare, the Directorate General of Health Services (DGHS) and the Directorate General of Family Planning (DGFP), deliver Expanded Programme on Immunization (EPI) and FP services, respectively. These directorates oversee separate service delivery points and provider cadres, with known coordination gaps. To integrate FP into EPI, the DGHS would need to revise job descriptions of health assistants, who provide immunization services, to allow FP service provision. Alternatively, the DGFP, would need to revise job descriptions of community-based family welfare assistants who provide FP services to encompass support to EPI sessions. These shifts would create displacement, including an expanded mandate with additional training and supervisory needs, commodity provision, increased provider workload, and possibly longer client wait times. Additionally, while about one-third of facilities had infrastructure allowing privacy during immunization visits, most facilities used public areas with few opportunities for private FP counseling or service provision. Figure 2 depicts the client journey map for this approach.

#### Strengthening Immediate PPFP for FTMs Delivering in Facilities

The Government of Bangladesh and Obstetrical and Gynecological Society of Bangladesh had both prioritized postpartum intrauterine devices. Many existing providers were trained in PPFP and provided integrated services, requiring minimal training and revisions to coordination mechanisms or existing job aids. Existing tools and processes would facilitate immediate PPFP delivery; PPFP counseling began in ANC, with preferences written in government-provided ANC cards reviewed during facility delivery. However, documentation of PPFP preferences was inconsistent. Counseling would require careful coordination and documentation to confirm FTMs’ PPFP intentions during intrapartum care. Lower facility delivery rates would limit immediate PPFP uptake.

#### Strengthening Predischarge Counseling for FTMs Delivering in Facilities

Many providers were trained in PPFP and provided integrated services, requiring minimal additional training or shifts to supervision and coordination processes. However, adding a tool to strengthen the predischarge counseling process risked expanding provider workload and client wait time due to additional tasks. Existing referral coordination (for follow-up PNC, including PPFP, and for other services before discharge of mother-baby dyads with a complication) was poor and a source of confusion for providers and clients. Lack of existing mechanisms documenting the condition of mother and baby at discharge complicated coordination.

#### Supporting Targeted Home Visits to FTMs

A community-level system for registering all pregnant women provided a platform for timely identification of FTMs beginning from pregnancy, although no existing mechanism would inform a family welfare assistant (FWA) of a facility or home delivery to prompt a postnatal HV. FWA job descriptions include HVs to all mothers. However, Bangladesh had not realized full HV coverage due to high FWA vacancy rates and infeasible targets, with each FWA assigned to visit approximately 600 households monthly, among other responsibilities. FWAs rarely received supportive supervision, and HVs were rarely observed.

### Final Selection and Mitigation Strategies

#### Final Selection of PPFP Approaches

In total, 18 members of the expanded design team completed the Google survey, scoring the 3 remaining approaches using the criteria determined in Module 1. On a synchronous call during Module 8, the core and expanded design teams discussed the highest- and lowest-scoring criteria for each approach assessed. The team discussed whether high-scoring items outweighed low-scoring items and identified strategies to mitigate risk reflected in lower-scoring items. Informed by these discussions and guided by the quantitative approaches (i.e., the VAT, Google survey), the core design team determined which approach(es) to move forward and identified mitigation strategies.

#### Final Approaches Selected Based on Potential for Impact and for Institutionalization

**FIGURE 3:**
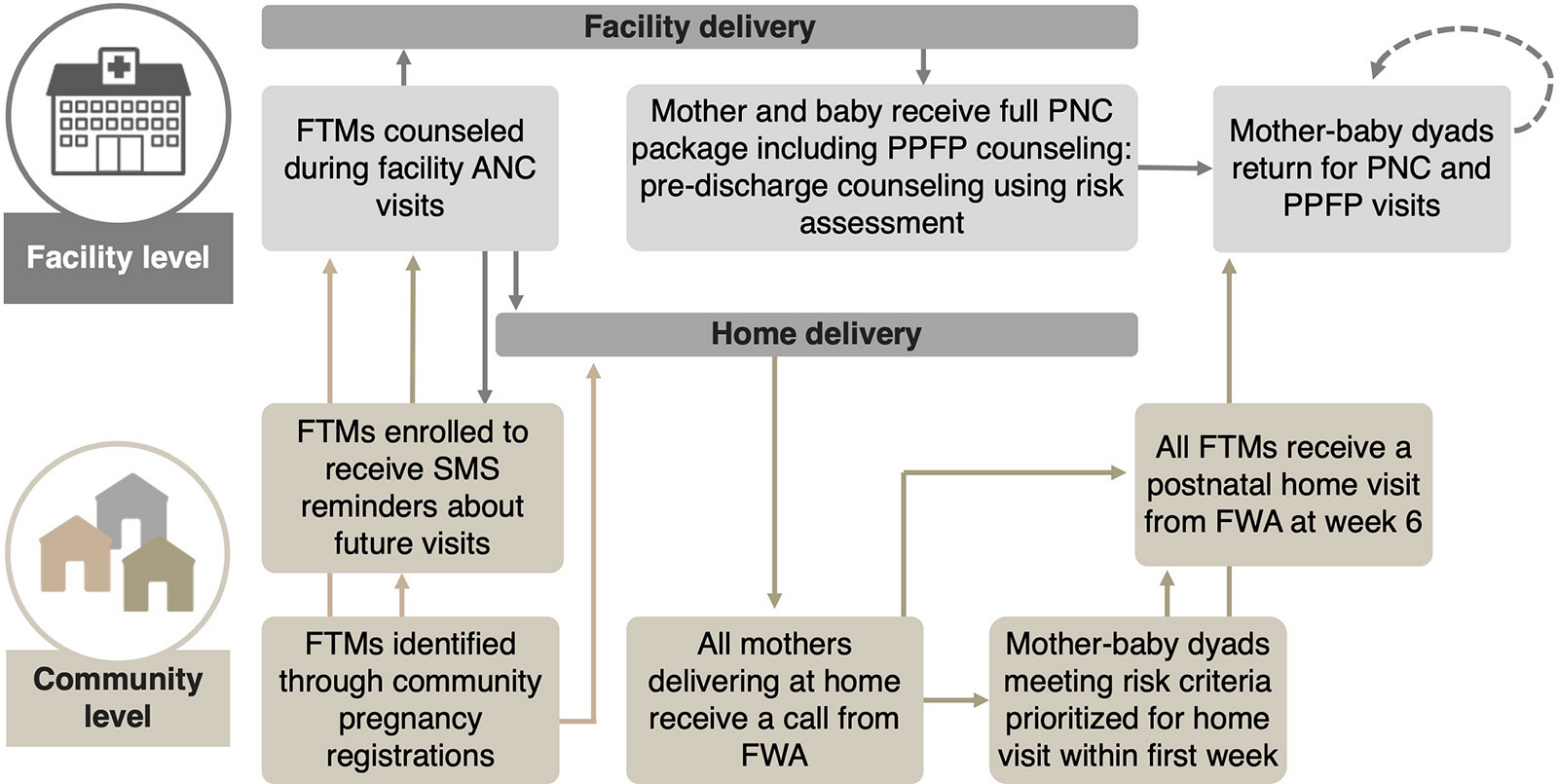
Final Combined Approaches Selected Based on Potential for Impact and for Institutionalization Abbreviations: ANC, antenatal care; FTM, first-time mother; FWA, family welfare assistant; PNC, postnatal care; PPFP, postpartum family planning; SMS, short message service.

The core design team selected the 3 remaining approaches (strengthening immediate PPFP, strengthening predischarge counseling, and supporting postnatal HVs) to be combined into a final approach, and developed mitigation strategies addressing identified risks. This approach aims to increase uptake of PPFP through strengthening the quality and responsiveness of facility-based antenatal and postnatal counseling and care (including predischarge counseling and care) to FTMs. At the community level, FWAs are supported to provide targeted postnatal HVs, prioritizing mother-baby dyads who are identified to have risk factors, including clinical and nonclinical (e.g., aged <20 years and primiparity).^30^ With the targeted PNC approach, FTMs, including those who deliver at home, are prioritized for FWA outreach. FWAs distribute a mother-baby booklet written for girls and young women becoming FTMs and their male partners, and a printed invitation card that invites FTMs to access RMNCH services, including PPFP, from the closest health facility. Figure 3 represents the journey map of the final combined approaches.

The final approach aims to increase PPFP uptake through strengthening the quality and responsiveness of facility-based counseling and care.

Combined, the selected approaches align with existing job descriptions and supervision processes, with mitigation measures to address identified gaps in workload, information, documentation, and supervision. The facility components entail minor adaptations to existing government approaches, tools, and processes. The introduction of the predischarge counseling approach will be carefully monitored to understand implications for workload and client flow. Introduction of a predischarge counseling approach also provides the opportunity to establish processes for documenting the status of mother-baby dyads at the time of discharge. The targeted approach to HVs is essential to mitigating challenges with HV coverages, to reaching FTMs regardless of place of delivery, and to engaging family.

## DISCUSSION

Many public health practitioners are most familiar with selecting approaches based on assessment of potential for impact. Yet any intervention is implemented within and inherently disrupts dynamic, complex health systems,^31^ which influence the potential to institutionalize. Our design and selection approach helped us to distinguish between potential for impact and potential for institutionalization—and underscores the need to move beyond designing for impact with only cursory thought to sustainability. Moreover, our experience shows that considerations of sustainability can be feasibly incorporated into design processes. With deeper assessment, we found that some initially promising approaches (i.e., PPFP/immunization integration) required system shifts infeasible to institutionalize or scale, or risked displacement of capacity. Our experience provides insights into key considerations for incorporating a systems lens in design processes.

### Stakeholder Engagement

The application of the VSM provided structure to guide stakeholders to identify the strengths and limitations of the platforms under consideration. Given that this was the first application of the VAT, we chose a qualitative approach to interpreting findings and to drawing actionable decisions. Ultimately, the design team made final decisions based on discussion and reflection on the facts gathered through the process, with decisions heavily influenced by identified deal-breakers that could not be mitigated. This underscores the importance of having the right stakeholders involved in the process so that their country experience, technical expertise, and knowledge of existing tools and platforms can help to distinguish a mitigatable risk from a deal-breaker. Close engagement with national stakeholders, as system experts and owners of institutionalization and scale, is key.^32^ While the information discussed through the process was known to many design team participants (e.g., the underresourced FWA cadre, and the coordination gaps between the Directorate General of Health Services and the Directorate General of Family Planning), the value of the exercise was in collectively synthesizing known information to pinpoint potential pitfalls and to identify proactive mitigation strategies.

The application of the VSM provided structure to guide stakeholders to identify strengths and limitations of platforms under consideration.

#### Systems Thinking in Design of Sustainable Approaches

The consideration of systems in general, or organizational functions more specifically, is not a panacea but provides useful tools to understand, analyze, and guide interventions for complex problems.^33^ Notably, the language of systems requires precision; we struggled with language as each approach considered involved different service delivery points, each a complex system. To our knowledge, this has been one of the first applications of VSM thinking to health systems strengthening in an LMIC context. Our experience provides several considerations for assessing the potential for institutionalization in design processes.

The established system inputs (expanded building blocks)^34^ emphasize *what* can be done, while the VSM-derived functions emphasize *how* it can be managed with minimal disruption to and adaptation of the existing systems. As elucidated through client journey maps, each service delivery point, including facilities and their service delivery units, is a micro-system in itself. Thus, strengthening a platform requires not only strengthening the core health system building blocks from the community to the highest levels of the health system, but also strengthening organizational functions at all levels. Visualizing a client’s journey illustrated the complexity of health systems. Beyond individual components, effective coordination and information sharing are required to work harmoniously.

We found that certain functions, namely service delivery interactions and coordination, elicited more robust discussion, and informed identification of viability deal-breakers and risk of displacement. In particular, we found necessitating formal shifts to job descriptions of underresourced cadres to be a key decision point, with such shifts beyond the scope and time frame of a donor-funded initiative and a deal-breaker overriding potential for impact. As in many LMICs, interventions relying on underresourced community health worker cadres in Bangladesh face threats to sustainability,^35^ underscoring the urgency of deeper investment in human resources for health.

#### Consideration of Context in Assessing Potential for Institutionalization

Our experience highlights the importance of contextual factors, including health system structure and capacity, in developing and adapting interventions and planning for sustainability.^36^ Notably, our findings around the potential for the institutionalization of the proposed approaches reflect context-specific factors; for example, while an approach integrating FP into immunization services was found to require unsustainable displacement within the Bangladeshi health system, others have scaled up integrated FP/immunization services in different contexts.^37^ In addition, while we did not identify significant risks within the supply chain and information systems functions in the specific context, these functions are vital and, notably, have limited success of PPFP models in other settings.^38^^,^^39^

Our experience highlights the importance of contextual factors, including health system structure and capacity, in planning for sustainability.

#### Considerations for Scalable FTM Approaches

Our process deepened insights into scalable approaches for increasing FTMs’ PPFP use that enhance existing government platforms, rather than unsustainable project-driven FTM outreach. While we designed approaches to address barriers to PPFP identified by FTMs, many barriers were not unique to FTMs (i.e., remaining at home during the postpartum period, limited decision-making power), and instead may reflect barriers experienced by all Bangladeshi mothers that may be aggravated by factors such as age, social norms, and lack of financial means.^40^ Given high rates of births to adolescents and young women in many LMICs,^41^ strengthening existing RMNCH platforms to better reach and meet the needs of the youngest mothers—while avoiding parallel platforms—is critical to achieving RMNCH goals.

Further efforts are needed to better understand the inputs needed to strengthen the underlying system functions along each step of a client’s journey, the associated measurement implications, and how most effectively to support health system actors to manage complexity. Further exploration of a systems thinking–informed means to identify and articulate displacement risks beyond the intervention design phase is warranted.

### Limitations

The adaptation to a virtual format due to COVID-19 eliminated the possibility of face-to-face workshops that may have enriched our discussions. However, the format of spacing design modules over a longer period of time offered unintended benefits, allowing for reflection and information gathering between synchronous discussions that would have been infeasible in a time-bound live workshop setting.

Health systems are by nature dynamic. While our application of the VSM proved valuable in understanding, analyzing, and guiding the design of the intervention, its use at an initial design phase does not (and we suggest should not) obviate the need for continued monitoring of unintended effects, including displacement to other services or demographic segments. Additionally, findings related to the potential to institutionalize each approach are specific to the unique contexts of the health systems in 1 country and are not intended to be generalizable.

Our use of the VSM at an initial design phase does not obviate the need for continued monitoring of unintended effects within a health system.

The VSM posits a fractal nature of organizations, with each level requiring viable functions similar to the whole system. While our tools probed on information and supply chain support systems, they remained cursory in the examination. We aimed to be as systematic as possible to build replicability in the design process, but it also included a substantial amount of iterative discussions, back-and-forth questioning, and even well-informed gut-checks, which all factored into decisions. While this critical collective engagement will always be necessary, our process could benefit from streamlining in subsequent applications.

In the context of a first implementation, we relied on a core team and an expanded design team of national stakeholders. We hope to see more expanded team members as central to the core team in future applications. Finally, the test of the viability of our efforts will be implementation, monitoring, evaluation, learning, and adaptive management.

## CONCLUSIONS

Our design process informed the selection of community- and facility-based approaches that had potential for both impact and institutionalization in context, guided us to move beyond approaches with limited institutionalization potential (i.e., deal-breakers), and informed proactive risk mitigation. Considerations of the feasibility of institutionalizing impactful approaches are underemphasized and require deeper attention in global health efforts, to the same level as the focus on the potential for an approach to produce desired impacts. Any approach has risks to sustainability; considering organizational functions in the design process can inform proactive mitigation approaches and ongoing monitoring efforts.

## Supplementary Material

GHSP-D-22-00023-supplement-1.pdf

GHSP-D-22-00023-supplement-3.pdf

GHSP-D-22-00023-supplement-2.pdf

## Notes

**Peer Reviewed**

**First published online:** October 21, 2022.
