# Supplementary material for: Designing for Impact and Institutionalization: Applying Systems Thinking to Sustainable Postpartum Family Planning Approaches for First-Time Mothers in Bangladesh
Source: Glob Health Sci Pract. 2022 Oct 31;10(5):e2200023. doi: 10.9745/GHSP-D-22-00023 (PMC9622282; doi:10.9745/GHSP-D-22-00023)
Supplement: GHSP-D-22-00023-supplement-1.pdf [file GHSP-D-22-00023-supplement-1.pdf]

Supplement to: Yahner M, Muriuki A, Mangieri A, Nitu S, Shafinaz S, Sarriot E. Designing for impact and institutionalization: applying systems thinking to sustainable postpartum family planning approaches for first-time mothers in Bangladesh. *Glob Health Sci Pract*. 2022;10(5):e2200023. https://doi.org/10.9745/GHSP-D-22-00023

Supplement 1. Selection Criteria for Approaches With Potential for Sustained Impact at Scale

denotes those drawn from ExpandNet guidance. §denotes those drawn from the Viable System Model

Potential for impact with first-time mothers

1. Likelihood the enhancement will improve utilization of PPFP for FTMs based on existing evidence, and/or
2. Can be clearly understood by implementers and clients
3. Has the potential to reach a significant % of the target population (FTMs), including younger (15-19) and older (20-24) FTMs
4. Is tailored to the specific needs and barriers of FTMs*
5. Appropriately considers social and gender norms influencing FTMs*

Potential for institutionalization

1. Contributes to and fits with National PPFP Action Plan*
2. Contributes to Government of Bangladesh Maternal Health Strategy*
3. Contributes to Health Population and Nutrition Sector Program*
4. Aligns with existing guidelines on PPFP service provision (or requires changes to guidelines that are feasible within the project's scope and mandate)
5. Builds on and also expands an existing community or facility service platform (does not create a new silo or separate system, schedule, curriculum, fragmented client experience)§
6. Required changes in human resources (responsibilities, task sharing, training, workload) are feasible within the project timeline and resources §
7. Supervision for the enhancement can build on existing supervision mechanisms and any needed adjustments are minimal§
