# Supplementary material for: Designing for Impact and Institutionalization: Applying Systems Thinking to Sustainable Postpartum Family Planning Approaches for First-Time Mothers in Bangladesh
Source: Glob Health Sci Pract. 2022 Oct 31;10(5):e2200023. doi: 10.9745/GHSP-D-22-00023 (PMC9622282; doi:10.9745/GHSP-D-22-00023)
Supplement: GHSP-D-22-00023-supplement-3.pdf [file GHSP-D-22-00023-supplement-3.pdf]

Supplement to: Yahner M, Muriuki A, Mangieri A, Nitu S, Shafinaz S, Sarriot E. Designing for impact and institutionalization: applying systems thinking to sustainable postpartum family planning approaches for first-time mothers in Bangladesh. *Glob Health Sci Pract*. 2022;10(5):e2200023. https://doi.org/10.9745/GHSP-D-22-00023

Supplement 3. Practical Application of Systems Thinking in Program Intervention Design: The Intervention Viability Assessment Tool

AIM

The purpose of this tool is to support designers and practitioners in assessing a potential program intervention to identify:

- its fit with the health system
- deal-breakers, pitfalls, and other issues for further discussion
- potential for impact

SECTION 1 – INTERVENTION DESCRIPTION

Purpose of table: to understand how the intervention will be implemented, by whom, where, how and the target client's journey from identification to referral.

- Entry point: where a service provider will interact with the client (e.g., household, facility) to provide information and/or services.
- Referral point: where a service provider may send the client for additional services related to the intervention (e.g., referral to a facility for postpartum family planning (PPFP) services after a household visit, referral for treatment of malnutrition from an immunization unit)

**1.** What is the proposed intervention? *(briefly explain the intervention)*	
**2.** What outcome does the intervention aim to achieve? *(state the intended outcome of the intervention)*	
**3.** Who is the target client of this intervention? *(state intended target client)*	
**4.** Is this intervention supported by existing evidence?	☐Yes<br>Briefly explain:<br>☐No<br>Briefly explain:
**5.** If this intervention is not supported by existing evidence, is it designed to generate evidence?	☐Yes<br>Briefly explain:<br>☐No<br>Briefly explain:
**6.** What tools will need to be developed or adapted for the implementation of this intervention (counselling, job aids, checklists etc.)?<br>*(list the key tools, if no new tools are needed indicate N/A)*	1.<br>2.<br>3.<br>4.

Supplement to: Yahner M, Muriuki A, Mangieri A, Nitu S, Shafinaz S, Sarriot E. Designing for impact and institutionalization: applying systems thinking to sustainable postpartum family planning approaches for first-time mothers in Bangladesh. *Glob Health Sci Pract*. 2022;10(5):e2200023. https://doi.org/10.9745/GHSP-D-22-00023

**7.** Does the intervention have additional data collection needs for monitoring beyond what is available in the national MoH health information system?	☐Yes<br>Explain if yes:<br>☐No
**ENTRY POINT**	
**8.** What is the primary entry point for this intervention where most target clients can be reached? *(specify unit within the facility)*	☐Household<br>☐Primary health facility<br>☐Secondary health facility<br>☐Tertiary health facility<br>☐ Other – Explain
**9.** Is this the lowest possible level where the intervention can be implemented without compromising quality?	☐Yes<br>☐No
**10.** How will the target clients be identified for specific focus at this entry point? *(briefly explain)*	
**11.** What service(s), if any, related to the intervention, are already being provided to the target client at this entry point? *(list services related to the intended outcome)*	1.<br>2.<br>3.<br>4.
**12.** Who will provide the service(s) proposed in this intervention, to the target client, at the entry point? *(name service provider(s)*	1.<br>2.<br>3.<br>4.
**13.** What capacity development will the service provider(s) at the entry point require to implement the intervention? *(list 4 key capacity building needs, if none is needed indicate N/A)*	1.<br>2.<br>3.<br>4.
**14.** Will these services proposed in the intervention affect the workload of the provider(s) at the entry point (e.g., new tasks, more clients, longer consultation time, etc.)?	☐Yes<br>☐No<br>Briefly explain:
**15.** Will the services proposed in the intervention affect client flow (e.g., confusion, longer waiting times, longer consultation times etc.)?	☐Yes<br>☐No<br>Briefly explain:
**REFERRAL POINT**	

Supplement to: Yahner M, Muriuki A, Mangieri A, Nitu S, Shafinaz S, Sarriot E. Designing for impact and institutionalization: applying systems thinking to sustainable postpartum family planning approaches for first-time mothers in Bangladesh. *Glob Health Sci Pract*. 2022;10(5):e2200023.
https://doi.org/10.9745/GHSP-D-22-00023

**16.** Will the intervention require the target client to be sent from the primary entry point to a referral point for provision of the services? *(If yes, please specify the unit within the referral point(s). If no, skip to section 2)*	☐Household<br>☐Primary health facility<br>☐Secondary health facility<br>☐Tertiary health facility<br>☐ Other – Explain
**17.** What service(s), related to the intervention, will be provided to the target client this referral point? *(list services to be provided)*	1.<br>2.<br>3.<br>4.
**18.** Who will provide the service(s) proposed in this intervention, to the target client, at the referral point? *(name service provider(s)*	1.<br>2.<br>3.<br>4.
**19.** What capacity development will the service provider(s) at the entry point require to implement the intervention? *(list 4 key capacity building needs, if none is needed indicate N/A)*	1.<br>2.<br>3.<br>4.
**20.** Will these services proposed in the intervention affect the workload of the provider at the referral point (e.g., new tasks, more clients, longer consultation time, etc.)?	☐Yes<br>☐No<br>Briefly explain:
**21.** Will the services proposed in the intervention affect client flow (e.g., confusion, longer waiting times, longer consultation time etc.)?	☐Yes<br>☐No<br>Briefly explain:

SECTION 2 – MANAGEMENT SYSTEMS

Purpose of table: To assess the viability of the intervention within the service delivery point by examining the essential elements required to provide the services required.

**Essential Service Delivery Operations**	
1. If the intervention is introduced, there are enough providers with the skills needed to conduct the activity(ies) and provide the service(s) proposed. (*check one)*	
**Entry point**<br>☐Totally agree<br>☐Agree<br>☐Disagree<br>☐Totally disagree	**Referral point**<br>☐Totally agree<br>☐Agree<br>☐Disagree<br>☐Totally disagree

Supplement to: Yahner M, Muriuki A, Mangieri A, Nitu S, Shafinaz S, Sarriot E. Designing for impact and institutionalization: applying systems thinking to sustainable postpartum family planning approaches for first-time mothers in Bangladesh. *Glob Health Sci Pract*. 2022;10(5):e2200023. https://doi.org/10.9745/GHSP-D-22-00023

2. If the intervention is introduced, the current supply of essential supplies and commodities is sufficient to meet any increase in use of services. (*check one)*	
**Entry point**<br>☐Totally agree<br>☐Agree<br>☐Disagree<br>☐Totally disagree<br>☐N/A	**Referral point**<br>☐Totally agree<br>☐Agree<br>☐Disagree<br>☐Totally disagree<br>☐N/A
3. The manager (or person in charge) of the service delivery point has all the required resources, information, and decision-making power to support the introduction and implementation of the intervention. *(Check one)*	
**Entry point**<br>☐Totally agree<br>☐Agree<br>☐Disagree<br>☐Totally disagree	**Referral point**<br>☐Totally agree<br>☐Agree<br>☐Disagree<br>☐Totally disagree
**Integration and coordination of services**	
4. Currently, the service providers where this intervention will be implemented only provide one unique type of service e.g. only provide immunization, only provide nutrition screening and referral. (*check one)*	
**Entry point**<br>☐Totally agree (they provide only one service)<br>☐Agree (they occasionally provide other service(s))<br>☐Disagree (they often provide other service(s))<br>☐Totally disagree (they already provide highly integrated services)	**Entry point**<br>☐Totally agree (they provide only one service)<br>☐Agree (they occasionally provide other service(s))<br>☐Disagree (they often provide other service(s))<br>☐Totally disagree (they already provide highly integrated services)
5. **If they provide only one type of service,** substantial service reorganization (change in client flow, infrastructure, number of staff, roles of staff etc.) is required to integrate the proposed intervention (*check one, explain your answer)*	

Supplement to: Yahner M, Muriuki A, Mangieri A, Nitu S, Shafinaz S, Sarriot E. Designing for impact and institutionalization: applying systems thinking to sustainable postpartum family planning approaches for first-time mothers in Bangladesh. *Glob Health Sci Pract*. 2022;10(5):e2200023. https://doi.org/10.9745/GHSP-D-22-00023

**Entry point**<br>☐Totally agree<br>☐Agree<br>☐Disagree<br>☐Totally disagree<br>Explain:	**Referral point**<br>☐Totally agree<br>☐Agree<br>☐Disagree<br>☐Totally disagree<br>Explain:
Skip to the next section (Immediate Oversight of Service Delivery) if **all** *services are provided at a single entry point, with no possible need for referral.*<br>6. If it may be necessary to send the client from the entry point to a referral point for provision of services, how well does coordination between providers in the two touchpoints work in order to support continuity of services? (*check one, explain your answer)*	☐Good, little confusion for providers and clients<br>☐Fair, some confusion for providers and clients<br>☐Poor, lots of confusion for providers and clients<br>☐There is currently no coordination between these two touch points<br>Explain:
7. What mechanisms are in place to improve coordination between the entry and referral points? (*list examples such as interdepartmental meetings, scheduling tools, supervision meetings between providers and community volunteers*	
**IMMEDIATE OVERSIGHT OF SERVICE DELIVERY**	
**Performance management, resource allocation and control**	
1. Who is the designated manager immediately overseeing service delivery (in-charge)? *(provide title and where she/he is located)*	Entry point<br>Referral point
2. If a service intervention is introduced, who will be responsible for giving service providers new and clear standard operating procedures for their work?	Entry point<br>Referral point
3. How often is the performance of providers and units reviewed and evaluated? *(e.g. monthly, quarterly, never)*	Entry point<br>Referral point
4. Who provides clinical supervision for providers responsible for the implementation of services and the intervention? *(provide title)*	Entry point<br>Referral point
5. How often is supportive supervision conducted? *(e.g. monthly, quarterly, never)*	Entry point<br>Referral point
**Planning ahead and Setting Rules and Policies**	

Supplement to: Yahner M, Muriuki A, Mangieri A, Nitu S, Shafinaz S, Sarriot E. Designing for impact and institutionalization: applying systems thinking to sustainable postpartum family planning approaches for first-time mothers in Bangladesh. *Glob Health Sci Pract*. 2022;10(5):e2200023. https://doi.org/10.9745/GHSP-D-22-00023

6. Who is responsible for ensuring that staff capacity is developed/are trained and updated on new guidelines and protocols? *(provide title)*	Entry point<br>Referral point
7. Who ensures that service staff have a clear plan for the weeks, months, and quarters ahead?	Entry point<br>Referral point
**SYSTEMS TO SUPPORT SERVICE DELIVERY**	
1 The management of commodities and supplies, for the intervention, is well-coordinated.	Entry point: ☐Yes ☐No<br>Referral point: ☐Yes ☐No
2 There is a system for assessing and evaluating how well commodities and supplies, needed for the intervention, are managed.	Entry point: ☐Yes ☐No<br>Referral point: ☐Yes ☐No
3 The management of information for decision making is well coordinated.	Entry point: ☐Yes ☐No<br>Referral point: ☐Yes ☐No

SECTION 3 – PROGRAMMATIC DECISIONS

Purpose of table: To make decisions based on the information provided in the sections above

1. What activities do you propose to strengthen management systems under the following areas: (*list 4 activities under each area)*	
Improving availability of staff, commodities, and supplies	1.<br>2.<br>3.<br>4.
Improving the provision of services	1.<br>2.<br>3.<br>4.
Improving coordination between entry and referral points	1.<br>2.<br>3.<br>4.
Improving supervision and support for health providers and units	1.<br>2.<br>3.<br>4.
2. What is currently being done (by the MoH, other partners) strengthen management systems under the areas mentioned above?	
3. What are the potential deal-breakers, or critical elements that would need to be in place for the intervention to work and are currently missing or weak?	

Supplement to: Yahner M, Muriuki A, Mangieri A, Nitu S, Shafinaz S, Sarriot E. Designing for impact and institutionalization: applying systems thinking to sustainable postpartum family planning approaches for first-time mothers in Bangladesh. *Glob Health Sci Pract*. 2022;10(5):e2200023. https://doi.org/10.9745/GHSP-D-22-00023

4. Based on the assessment, this intervention can be **introduced into the health system and scaled up in this context.** *(Explain your answer)*	☐Totally agree<br>☐Agree<br>☐Disagree<br>☐Totally disagree

Do you have any overall questions or concerns about this intervention that were not addressed in the questions above? Please note them here.
