# Supplementary material for: Designing for Impact and Institutionalization: Applying Systems Thinking to Sustainable Postpartum Family Planning Approaches for First-Time Mothers in Bangladesh
Source: Glob Health Sci Pract. 2022 Oct 31;10(5):e2200023. doi: 10.9745/GHSP-D-22-00023 (PMC9622282; doi:10.9745/GHSP-D-22-00023)
Supplement: GHSP-D-22-00023-supplement-2.pdf [file GHSP-D-22-00023-supplement-2.pdf]

Supplement to: Yahner M, Muriuki A, Mangieri A, Nitu S, Shafinaz S, Sarriot E. Designing for impact and institutionalization: applying systems thinking to sustainable postpartum family planning approaches for first-time mothers in Bangladesh. *Glob Health Sci Pract*. 2022;10(5):e2200023. https://doi.org/10.9745/GHSP-D-22-00023

Supplement 2. VSM Systems Mapped to Health System

VSM Systems	Description	Considerations for Health Systems
System 1: Operations	"Primary organizational activities", delivering goods/services to "clients". Operations include clients, geography, service time, technology. Operations must have sufficient decision-making autonomy to address clients' demands and problems.	Frontline delivery of different facility- and community-level curative and preventive services (nutrition, child health, maternal health, newborn health, reproductive health, immunization).
System 2: Coordination	Tools and mechanisms, including schedules, standard operating procedures, mutually-developed rules and language to resolve conflict (space, time, resources, clients) between different operations (service units), as close to service units as possible. Strong coordination reduces higher-level management burden.	Coordinated use of shared resources, e.g., clinic space, vehicles (supervision and outreach), human resources tasked with delivering services to different groups at different times and locations.
Support activities	Some activities require operations not oriented toward the client environment of the organization, but in support of the primary activities of the organization. Support activities must be structured as a viable system with their own defined Operations, Coordination, and Management Functions to be viable.	Needed support activities include supply chain management, information technology infrastructure, and human resources management. Operations (System 1) face the environment of service providers (facility or community-based), requiring coordination and System 3 management.
**Strategy and Management Functions**		
System 3: Operational Control (Resources and Performance), aka Supervision, Management, Oversight, Control	Managers must resource and support processes of Operations, balancing resources and performance. This requires organizational skills, participatory planning, and interpersonal communication. With insufficient coordination, System 3 may be engaged to resolve performance gaps (e.g. service quality). When primary units lack sufficient autonomy, System 3 management may engage in micro-management and inefficient time use.	Facility in-charges are usually a first layer of delivery management. District-level structures (health districts, local government) serve this function at the next level.
System 3*: Monitoring	Monitoring requires that managers receive reliable signals confirming or correcting information from primary service providers, and guaranteeing that managers understand the services that providers deliver. Monitoring must be carefully tailored to avoid overwhelming management with data better used at Operations level, and to respect the autonomy of these operations level.	Monitoring requirements are in principle well-understood, but often under-resourced. Routine service data feed into HMIS, but rules for aggregation (reducing data complexity) and appropriate data use are frequent challenges.
System 4: Anticipating/ Development (aka Higher-level Management/Planning)	Viable organizations need mechanisms to understand and anticipate changes in the dynamic environment to prepare for and adapt to changes.	Health systems may not specify where this function is served, except at the highest levels. When service delivery structures rely on central systems and when autonomy is lacking, risks include time lags between identifying emerging changes (e.g., changing

Supplement to: Yahner M, Muriuki A, Mangieri A, Nitu S, Shafinaz S, Sarriot E. Designing for impact and institutionalization: applying systems thinking to sustainable postpartum family planning approaches for first-time mothers in Bangladesh. *Glob Health Sci Pract*. 2022;10(5):e2200023. https://doi.org/10.9745/GHSP-D-22-00023

		populations, new epidemiological threats, etc.) and response.
System 5: Policy (aka Policy and Governance)	Encompasses official policy but also broader governance, setting rules to structure and allow debate to adapt strategies, ensuring cohesion and coherence of organizational efforts and values.	Health system governance is recognized through health systems frameworks, but often associated with central-level "health policy" functions. System 5 functions can also be required at subnational and frontline levels, establishing each level's values and normative rules.
